# Items from Our Correspondents

**Published:** 1858-02

**Authors:** 


### ITEMS FROM OUR CORRESPONDENTS.

AURORA, Feb. 2d, 1858.

PROF. N. S. DAVIS, M. D.,

DEAR SIR,—On January 16th, the regular practitioners of medicine of this city met at Dr. D. W. Young's office and organized a Medical Society, which is called "THE AURORA CITY MEDICAL SOCIETY."

The officers of the society are as follows:

President, . . . . . . . . . . P. A. Allaire, M. D.
Vice-President, . . . . . . . O. D. Howell, M. D.
Secretary, . . . . . . . . . . D. W. Young, M. D.
Treasurer, . . . . . . . . . . A. Hard, M. D.

The society meets on the first Monday of each month, and has adopted the code of medical ethics of the Illinois State Medical Society for its government.

D. W. YOUNG, *Secretary.*

---

FULTON CITY, ILLINOIS.

MR. EDITOR:

DEAR SIR,—I notice in this July number of the *Journal*, an explanation of the signs and symptoms of pregnancy, by W. P. Montgomery, from the second London edition, etc. In his chapter he gives all or many of the supposed well known signs of pregnancy, all of which, except two, he says, are deceptive. He, therefore, gives two as proof positive of this condition, viz: the pulsation of the fœtal heart, and motion or movements of the child; but these are signs given by the child, and I would inquire, is there no positive proof furnished by the mother? In answer, I would say there is a sign provided by the mother, as positive, and more so, than any presented by the child. I never look for either of the two positive proofs he speaks of, for I have a more sure way. Yes, a more positive sign, that is a papula, or pimples on the edges of the os tincæ or mouth of the womb, and when this condition is present, I never hesitate to declare, on oath or otherwise, that the woman is pregnant; and yet in no case have I been deceived, and I am sure I never shall be, if the os tincæ is in other respects healthy. There is a lady near me, who asked my opinion about nine months ago; the condition of os tincæ being as above described, I declared her to be in the family way, and in February last she furnished the proof; and I might furnish other cases, but I presume most of the profession are well acquainted with this my test; if I supposed they were not I would be more minute in describing it. If I were to decide in a case of life or death, I should rely more upon this sign furnished by the mother, than any furnished by the child.

Respectfully,

JOHN EDDY, M. D.

[Will Dr. Eddy have the kindness to forward a full description of this papular eruption on the os tincæ, whether discernible by the touch, or only to be seen through the speculum? We ask this, because it is undoubtedly new, and if he is not mistaken in its infallibility, promises to be exceedingly valuable.—ED.]

